# Secondary pulmonary infection and co-infection in elderly COVID-19 patients during the pandemics in a tertiary general hospital in Beijing, China

**DOI:** 10.3389/fmicb.2023.1280026

**Published:** 2023-10-12

**Authors:** Chaoe Zhou, Yaping Jiang, Liying Sun, Haixia Li, Xinmin Liu, Lei Huang

**Affiliations:** ^1^Department of Geriatrics, Peking University First Hospital, Beijing, China; ^2^Department of Clinical Laboratory, Peking University First Hospital, Beijing, China

**Keywords:** COVID-19, elderly, co-infection, secondary infection, Carbapenem-resistant Gram-negative bacilli

## Abstract

**Background:**

Most people are infected with COVID-19 during pandemics at the end of 2022. Older patients were more vulnerable. However, the incidence of secondary bacterial, fungal or viral pulmonary infection and co-infection is not well described in elderly hospitalized COVID-19 patients.

**Methods:**

We retrospectively reviewed the medical records of all elderly (≥65 years) hospitalized patients with laboratory-confirmed COVID-19 from December 1, 2022 to January 31, 2023. Demographics, underlying diseases, treatments, and laboratory data were collected. Univariate and multivariate logistic regression models were used to explore the risk factors associated with secondary bacterial, fungal or viral pulmonary infection and co-infection.

**Results:**

A total of 322 older patients with COVID-19 were enrolled. The incidence of secondary bacterial, fungal or viral pulmonary infection and co-infection was 27.3% (88/322) and 7.5% (24/322), respectively. The overall in-hospital mortality of all patients was 32.9% (106/322), and the in-hospital mortality among patients who acquired with secondary pulmonary infection and co-infection was 57.0% (57/100). A total of 23.9% (77/322) of patients were admitted to ICU within 48 h of hospitalization. The incidence of secondary pulmonary infection and co-infection among patients admitted to the ICU was 50.6% (39/77) and 13.0% (10/77), respectively. The overall in-hospital mortality of ICU patients was 48.1% (37/77), and the in-hospital mortality of ICU patients acquired with secondary pulmonary infection and co-infection was 61.4% (27/44). A total of 83.5% (269/322) of the included patients received empirical antibiotic therapy before positive Clinical Microbiology results. Influenza A virus (the vast majority were the H3N2 subtype) was the most common community acquired pathogen for co-infection. While *A. baumannii*, *K. pneumoniae*, and *P. aeruginosa* were the common hospital acquired pathogens for co-infection and secondary pulmonary infection. The incidence of Carbapenem-resistant Gram-negative bacilli (CR-GNB) infections was high, and the mortality reached 76.9%. Predictors of secondary pulmonary infection and co-infection were ICU admission within 48 h of hospitalization, cerebrovascular diseases, critical COVID-19, and PCT > 0.5 ng/mL.

**Conclusion:**

The prognosis for elderly hospitalized COVID-19 patients with secondary pulmonary infection or co-infection is poor. The inflammatory biomarker PCT > 0.5 ng/mL played an important role in the early prediction of secondary pulmonary infection and co-infection in COVID-19 patients.

## 1. Introduction

Coronavirus disease 2019 (COVID-19), was caused by severe acute respiratory syndrome coronavirus 2 (SARS-CoV-2). It was first identified in December 2019 in Wuhan, China, then spread rapidly worldwide. By May 17, 2023, the WHO reported a total of 766,440,796 confirmed cases of COVID-19 globally, including 6,932,591 deaths.^1^ In December 2022, the Chinese government announced that COVID-19 patients did not need to be quarantined. Within the next 2 months, a significant proportion of people were infected with SARS-CoV-2, especially elderly individuals. It posed formidable medical challenges for healthcare systems and clinicians.

Secondary bacterial, fungal or viral pulmonary infection and co-infection are a common and dangerous complication of COVID-19. A previous study showed that the incidence of co-infection and secondary infection in COVID-19 patients was generally low, but the incidence in Intensive Care Unit (ICU) patients was relatively high (Langford et al., 2020). It was significantly associated with poor clinical outcomes. According to existing report, 50% of COVID-19 deaths experienced secondary bacterial infection (Li et al., 2020). Several studies indicated that the majority of COVID-19 patients (>90%) received empirical antibiotics, which increased the risk of Multi-Drug Resistance (MDR) infection (Du et al., 2020; Wang Z. et al., 2020; Wu et al., 2020). International guidelines recommended empirical antibiotic therapy only for the possible occurrence of bacterial pneumonia in critically ill patients with COVID-19 (Poston et al., 2020).

Understanding the incidence of secondary bacterial, fungal or viral pulmonary infection and co-infection, and common pathogens in COVID-19 patients is crucial for appropriate antimicrobial therapy and improving outcomes. However, these data in older patients with COVID-19 have not been well characterized yet. There was only one relevant study on older patients (Wang L. et al., 2020). The objective of this study was to determine the incidence of secondary bacterial, fungal or viral pulmonary infection and co-infection in elderly COVID-19 patients, and to identify common pathogens. We also identified predictors independently associated with the development of infection.

## 2. Materials and methods

### 2.1. Study design and participants

We conducted a single-center, retrospective, observational study. It included all elderly (age ≥65 years) patients infected with COVID-19 who were consecutively hospitalized at Peking University First Hospital between December 2022 and January 2023 in Beijing, China. The exclusion criteria were as follows: (a) some key information was missing from the medical record; and (b) pathogens isolated from non-respiratory tract or non-bloodstream sources. The diagnosis of COVID-19 was made through Reverse Transcriptase real-time fluorescence Polymerase Chain Reaction (RT-PCR) in all cases from nasal or pharyngeal swabs. The primary outcome was the incidence of secondary bacterial, fungal or viral pulmonary infection and co-infection. We also assessed the risk factors associated with secondary bacterial, fungal or viral pulmonary infection and co-infection, and the impact of infection on clinical outcomes. Hospitalized patients with COVID-19 developed respiratory failure, required mechanical ventilation, was in shock or combined with other organ failure, they should be admitted to ICU for further treatment.

### 2.2. Data collection

Extracting clinical data from the electronic medical records, including patient demographics, underlying diseases, laboratory tests, clinical symptoms, treatments, microbiological results (blood culture, respiratory specimen culture, specific real-time fluorescence PCR test, urinary antigen test, and antimicrobial susceptibility testing), and clinical outcomes (in-hospital mortality, length of hospital stay, ICU admission, length of ICU stay, invasive mechanical ventilation, and the duration of invasive mechanical ventilation). All above data were entered into the computerized database for further statistical analyses.

### 2.3. Definitions

The severity of COVID-19 was defined according to the Chinese management guidelines for COVID-19 (version 10.0).^2^ In brief, the disease was defined as critical if the patient met one of the following conditions: respiratory failure occurred and required mechanical ventilation, shock, or other organ dysfunction requiring ICU monitoring and treatment. The definition of septic shock was based on the 2016 Third International Consensus Definition for Sepsis and Septic Shock (Singer et al., 2016). Pulmonary infection included either (a) co-infection, defined as patients with confirmed COVID-19 disease with other simultaneously co-infected pathogens (<48 h), or (b) secondary infection, defined as patients infected by other new pathogens after 48 h of hospital admission (Lansbury et al., 2020). Bloodstream infections were defined as at least one bottle of positive blood culture for a likely pathogen or ≥2 bottles of positive blood cultures for common skin colonizers (for example, Coagulase-negative *Staphylococci*, *Bacillus* spp., *Diphtheroids*, *Propionibacterium* spp., *Viridans group Streptococci*) (Ripa et al., 2021). Interleukin-6 (IL-6) or Janus kinase (JAK) inhibitors were tocilizumab and baricitinib.

### 2.4. Pathogen identification and antimicrobial susceptibility testing

The attending physicians decided to order microbiological tests for patients with suspected infection, and the clinical microbiologist handled with standard microbiological procedures. The cultured bacterial single colony was identified to species level by matrix-assisted laser desorption/ionization time of flight mass spectrometry (MALDI-TOF) (Croxatto et al., 2012). Antimicrobial susceptibility testing was performed using VITEK 2 Compact automated system (Biomerieux, France) according to the manufacturer’s instructions. Carbapenem-resistant bacteria were characterized by resistance to at least one kind of carbapenem (meropenem, imipenem, or ertapenem). The Minimal Inhibitory Concentrations (MICs) were determined and classified to susceptible, resistant, or intermediate according to breakpoints established by the Clinical and Laboratory Standards Institute (CLSI) (CLSI M100 31th) (Clinical and Laboratory Standards Institute, 2021).

### 2.5. Statistical analysis

Descriptive statistics included the median [interquartile range (IQR)] of continuous variables and n (%) of categorical variables. Mann-Whitney *U*-tests, χ2 tests or Fisher’s exact tests were used to compare the differences between infection and non-infection, the survivors and non-survivors. Univariate and multivariate logistic regression models were used to explore the risk factors associated with infection. The variables identified as significant (*p* < 0.05) in the univariate analysis entered into the multivariate logistic regression models. Some laboratory findings, including serum ferritin, IL-6, lactate dehydrogenase, high-sensitivity cardiac troponin I, creatine kinase-MB, and D-dimer may not be available in some patients, therefore, only lymphocyte counts, procalcitonin (PCT), and hyper C-reactive protein were entered into the multivariate logistic regression models. All reported *p*-values were double-tailed. All analyses were performed using SPSS version 26 (IBM Corp, Armonk, NY, USA).

## 3. Results

There was a total of 516 inpatients with RT-PCR confirmed COVID-19 infection, 194 inpatients were excluded (20 inpatients were non-respiratory infection, and 174 patients were aged <65 years), thus, the remaining 322 elderly patients were eventually included for further analyses. A total of 65.5% of included patients were male. The median age was 81.0 (72.0-87.0) years. A total of 59.3% of patients had a Charlson comorbidity index ≥3. Hypertension (67.1%) was the most common underlying disease, followed by chronic cardiovascular diseases (52.5%), chronic respiratory diseases (38.5%), cerebrovascular diseases (37.3%), and diabetes mellitus (37.0%). The most common symptom of COVID-19 infection was fever (81.1%), followed by cough (72.7%) and expectoration (68.0%) (Table 1). A total of 23.9% (77/322) of these patients were admitted to the ICU within 48 h of hospitalization. A total of 36.3% (117/322) of patients were classified as critical COVID-19. A total of 83.5% (269/322) of patients received empirical antibiotic therapy prior to the first positive culture. A total of 69.6% (224/322) of patients received antiviral treatments (paxlovid or azvudine) (Table 1).

**TABLE 1:** Summary of clinical features and laboratory results between infection and non-infection groups.

Variables	Total (*n* = 322)	Infection (*n* = 100)	Non-infection (*n* = 222)	*p*
Gender, male, *n* (%)	211 (65.5%)	66 (66.0%)	145 (65.3%)	0.905
Age, median (IQR)	81.0 (72.0–87.0)	83.0 (75.3–88.0)	79.0 (71.0–86.0)	0.002
ICU admission within 48 h of hospitalization, *n* (%)	77 (23.9%)	44 (44.0%)	33 (14.9%)	<0.001
**Underlying diseases, *n* (%)**				
Hypertension	216 (67.1%)	70 (70.0%)	146 (65.8%)	0.454
Diabetes mellitus	119 (37.0%)	35 (35.0%)	84 (37.8%)	0.625
Chronic cardiovascular diseases	169 (52.5%)	61 (61.0%)	108 (48.6%)	0.040
Chronic respiratory diseases	124 (38.5%)	36 (36.0%)	88 (39.6%)	0.535
Chronic liver diseases	26 (8.1%)	11 (11.0%)	15 (6.8%)	0.196
Chronic kidney diseases	79 (24.5%)	24 (24.0%)	55 (24.8%)	0.881
Cerebrovascular diseases	120 (37.3%)	47 (47.0%)	73 (32.9%)	0.015
Peripheral vascular diseases	82 (25.5%)	30 (30.0%)	52 (23.4%)	0.210
Hemopathy	20 (6.2%)	5 (5.0%)	15 (6.8%)	0.546
Connective tissue diseases	13 (4.0%)	1 (1.0%)	12 (5.4%)	0.121
Peptic ulcer	26 (8.1%)	9 (9.0%)	17 (7.7%)	0.682
Hemiplegia	9 (2.8%)	5 (5.0%)	4 (1.8%)	0.213
Malignancy	87 (27.0%)	24 (24.0%)	63 (28.4%)	0.413
Charlson comorbidity index ≥3	191 (59.3%)	59 (59.0%)	41 (41.0%)	0.938
**Exposure history, *n* (%)**				
Smoking	97 (30.1%)	32 (32.0%)	65 (29.3%)	0.622
Drinking	49 (15.2%)	14 (14.0%)	35 (15.8%)	0.683
Vaccination	32 (9.9%)	10 (10.0%)	22 (9.9%)	0.308
Previous glucocorticoid use	24 (7.5%)	3 (3.0%)	21 (9.5%)	0.070
Previous invasive procedure within 3 months before hospital admission	52 (16.1%)	20 (20.0%)	32 (14.4%)	0.208
Empirical use of antibiotics before the first positive culture	269 (83.5%)	96 (96.0%)	173 (77.9%)	<0.001
**Clinical symptoms, *n* (%)**				
Fever	261 (81.1%)	90 (90.0%)	171 (77.0%)	0.006
Sore throat	48 (14.9%)	12 (12.0%)	36 (16.2%)	0.326
Cough	234 (72.7%)	76 (76.0%)	158 (71.2%)	0.368
Sputum	219 (68.0%)	75 (75.0%)	144 (64.9%)	0.071
Fatigue	93 (28.9%)	21 (21.0%)	72 (32.4%)	0.036
Myalgias	33 (10.2%)	11 (11.0%)	22 (9.9%)	0.765
Diarrhea	37 (11.5%)	14 (14.0%)	23 (10.4%)	0.343
Thoracalgia	11 (3.4%)	3 (3.0%)	8 (3.6%)	1.000
Respiratory failure	77 (23.9%)	45 (45.0%)	32 (14.4%)	<0.001
**Laboratory findings, median (IQR)**				
Write blood count × 10^9 per L	6.7 (4.7–9.8)	7.7 (5.2–11.7)	6.3 (4.3–9.1)	0.007
Neutrophil count × 10^9 per L	5.2 (3.3–8.5)	6.5 (4.2–9.9)	4.8 (2.8–7.6)	0.001
Lymphocyte count × 10^9 per L	0.6 (0.4–0.9)	0.5 (0.3–0.8)	0.6 (0.4–1.0)	0.007
Lymphocyte count < 0.8 × 10^9 per L, n (%)	199 (61.8%)	72 (72.0%)	127 (57.2%)	0.017
Plate count × 10^9 per L	155.0 (111.0–214.0)	145.0 (104.0–196.0)	158.0 (117.3–225.3)	0.058
Neutrophilic granulocyte percentage	81.0 (69.6–88.7)	86.2 (77.8–91.7)	79.0 (66.2–86.1)	<0.001
Lymphocyte percentage	10.2 (5.1–17.3)	6.7 (3.9–12.8)	11.9 (6.2–19.0)	<0.001
Prothrombin time, s	11.9 (11.3–13.0)	12.4 (11.6–14.7)	11.8 (11.2–12.8)	<0.001
Activated partial thromboplastin time, s	31.1 (28.3–34.4)	32.5 (28.9–37.2)	30.8 (27.9–33.2)	0.002
D-dimer, mg/L	0.6 (0.3–1.2)	0.6 (0.4–2.3)	0.5 (0.3–1.1)	0.012
High-sensitivity cardiac troponin I, ng/L	20.4 (9.6–59.4)	39.8 (18.0–145.3)	15.1 (7.8–36.8)	<0.001
Creatine kinase-MB, mg/mL	1.9 (1.1–3.8)	2.5 (1.3–7.2)	1.7 (1.0–2.8)	<0.001
Creatinine, μmol/L	91.3 (72.0–158.6)	106.7 (75.5–185.4)	89.8 (69.1–140.3)	0.057
Lactic dehydrogenase, U/L	271.0 (204.5–384.5)	311.0 (218.5–455.0)	255.0 (192.0–360.0)	0.001
Serum ferritin, ng/mL	617.1 (261.6–1053.8)	912.4 (399.7–1091.8)	517.4 (172.6–739.8)	0.033
IL-6, pg/mL,	49.7 (19.6–124.7)	65.7 (30.4–192.1)	41.1 (14.8–91.5)	0.001
PCT, ng/mL	0.2 (0.1–0.8)	0.5 (0.2–1.7)	0.1 (0.1–0.5)	<0.001
PCT > 0.5 ng/mL, *n* (%)	93 (28.9%)	48 (48.0%)	45 (20.3%)	<0.001
Hyper C-reactive protein, mg/dL	65.9 (24.5–113.1)	88.5 (44.4–135.3)	54.8 (19.0–105.0)	<0.001
**Critical COVID-19**	117 (36.3%)	65 (65.0%)	52 (23.4%)	<0.001
**Antiviral treatment, *n* (%)**	224 (69.6%)	81 (81.0%)	143 (64.4%)	0.003
Paxlovid	132 (41.0%)	58 (58.0%)	74 (33.3%)	<0.001
Azvudine	131 (40.7%)	45 (45.0%)	86 (38.7%)	0.290
**Immunomodulatory treatment**				
IL-6 or JAK inhibitors, *n* (%)	38 (11.8%)	20 (20.0%)	18 (8.1%)	0.002
Immunoglobulin therapy, *n (%)*	33 (10.2%)	19 (19.0%)	14 (6.3%)	0.001
Glucocorticoid treatment, *n* (%)	150 (46.6%)	64 (64.0%)	86 (38.7%)	<0.001
Time from onset of symptom to start of glucocorticoid treatment, days, median, (IQR)	8.0 (5.0–12.0)	8.5 (6.0–12.0)	8.0 (4.0–12.0)	0.593
Glucocorticoid treatment duration, median, (IQR)	6.0 (3.0–10.0)	5.0 (3.0–9.8)	7.0 (3.8–10.0)	0.526
ECMO, *n* (%)	3 (0.9%)	2 (2.0%)	1 (0.5%)	0.476
CRRT, *n* (%)	16 (5.0%)	10 (10.0%)	6 (2.7%)	0.005
**Anticoagulant therapy**	167 (51.9%)	70 (70.0%)	97 (43.7%)	<0.001
**Outcomes**				
ICU admission in hospitalization, *n* (%)	97 (30.1%)	57 (57.0%)	40 (18.0%)	<0.001
Length of ICU stay, days, median (IQR)	9.5 (6.0–17.3)	12.0 (8.0–25.0)	7.0 (3.5–10.0)	<0.001
Mechanism ventilation, *n* (%)	57 (17.7%)	42 (42.0%)	15 (6.8%)	<0.001
Duration of mechanism ventilation, hours, median (IQR)	122.0 (40.0–331.0)	211.5 (75.5–495.0)	38.0 (3.0–58.0)	<0.001
Septic shock, *n* (%)	55 (17.1%)	43 (43.0%)	12 (5.4%)	<0.001
In-hospital mortality, *n* (%)	106 (32.9%)	57 (57.0%)	49 (22.1%)	<0.001
Length of hospital stay from exposure to SARS-COV-2, days, median (IQR)	13.0 (7.0–21.0)	18.5 (11.0–29.0)	10.0 (6.0–18.0)	<0.001
Length of hospital stay, median (IQR)	16.0 (8.0–29.0)	23.0 (14.3–45.0)	14.0 (7.0–23.3)	<0.001

IQR, range interquartile; IL-6, interleukin-6; PCT, procalcitonin; JAK, Janus kinase; ECMO, extracorporeal membrane oxygenation; CRRT, continuous renal replacement therapy.

### 3.1. The clinical characteristics between bacterial infection and non-infection

**FIGURE 1:**
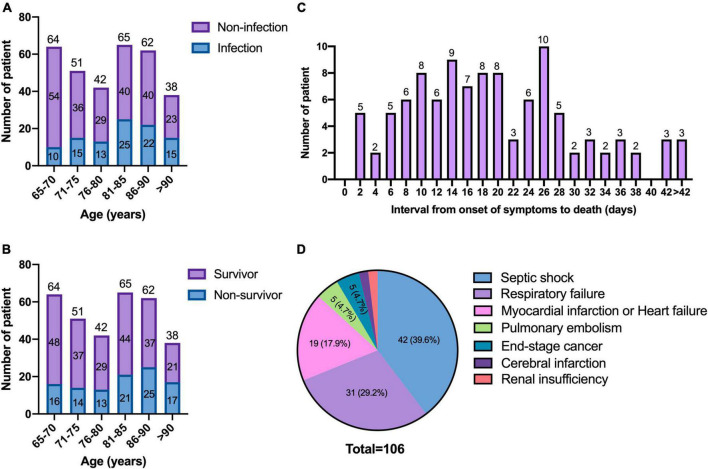
Age distribution and the causes of mortality. **(A)** Age distribution of patients between infection and non-infection; **(B)** age distribution of patients between the survivors and non-survivors; **(C)** interval from onset of symptoms to death of patients; **(D)** causes of mortality.

The overall proportion of COVID-19 patients with secondary bacterial, fungal or viral pulmonary infection and co-infection was 31.1% (100/322). The distribution of age between patients with and without infection is shown in Figure 1A, which increased in parallel with age (*p* = 0.002).

The clinical characteristics between the two groups were compared (Table 1). Patients with chronic cardiovascular diseases and cerebrovascular diseases were more vulnerable to infection. In addition, Patients admitted to ICU within 48 h of hospitalization (44.0 vs. 14.9%, *p* < 0.001), classified as critical COVID-19 (65.0 vs. 23.4%, *p* < 0.001), received invasive mechanical ventilation (42.0 vs. 6.8%, *p* < 0.001) were more likely to be infected. More infected patients suffered from septic shock (43.0 vs. 5.4%, *p* < 0.001). Infected patients had higher in-hospital mortality (57.0 vs. 22.1%, *p* < 0.001), higher proportion of ICU admission (57.0 vs. 18.0%, *p* < 0.001), longer length of hospital stay (median days 23.0 vs. 14.0, *p* < 0.001), and longer length of ICU stay (median days 12.0 vs. 7.0, *p* < 0.001) than patients without infection.

Multivariate logistic regression analysis showed patients admitted to ICU within 48 h of hospitalization (*p* < 0.001, OR = 2.880, 95% CI, 1.520–5.457), with cerebrovascular diseases (*p* = 0.022, OR = 2.031, 95% CI, 1.109–3.719), classified as critical COVID-19 (*p* < 0.001, OR = 4.160, 95% CI, 2.245–7.707), and PCT > 0.5 ng/mL (*p* = 0.046, OR = 1.989, 95% CI, 1.014–3.902) were independent risk factors of secondary bacterial, fungal or viral pulmonary infection and co-infection (Table 2).

**TABLE 2:** Independent risk factors for pulmonary infection in COVID-19 patients.

	Univariate		Multivariate	
**Variables**	**OR (95% CI)**	***p***	**OR (95% CI)**	***p***
ICU admission within 48 h of hospitalization	4.50 (2.620–7.729)	<0.001	2.880 (1.516–5.449)	0.001
Chronic cardiovascular diseases	1.651 (1.021–2.669)	0.040	1.111 (0.605–2.038)	0.735
Cerebrovascular diseases	1.810 (1.117–2.932)	0.015	2.031 (1.109–3.719)	0.022
Empirical use of antibiotics before the first positive culture	6.798 (2.380–19.411)	<0.001	3.626 (0.786–16.726)	0.099
Critical COVID-19	6.071 (3.627–10.162)	<0.001	4.160 (2.245–7.707)	<0.001
PCT > 0.5 ng/mL	3.156 (1.872–5.318)	<0.001	1.989 (1.014–3.902)	0.046
Lymphopenia*	1.863 (1.116–3.109)	0.017	0.966 (0.510–1.832)	0.916
Hyper C-reactive protein	0.994 (0.991–0.998)	<0.001	1.000 (0.995–1.005)	0.973

*Refers to a lymphocyte count <0.8 × 10^9 per.

### 3.2. Incidence of secondary pulmonary infection and co-infection

The incidence of secondary bacterial, fungal or viral pulmonary infection and co-infection was 27.3% (88/322) and 7.5% (24/322), respectively. A total of 23.9% (77/322) of patients were admitted to ICU within 48 h of hospitalization. The incidence of secondary pulmonary infection and co-infection among patients admitted to the ICU was 50.6% (39/77) and 13.0% (10/77), respectively. In this study, 83.5% (269/322) of patients received empirical antibiotic therapy before positive Clinical Microbiology results, predominantly carbapenems (*n* = 112), followed by cefoperazone/sulbactam (*n* = 104), cephalosporin (*n* = 72), moxifloxacin (*n* = 46), piperacillin tazobactam (*n* = 30). In particular, 124 patients received empirical therapy with more than one antibiotic.

**FIGURE 2:**
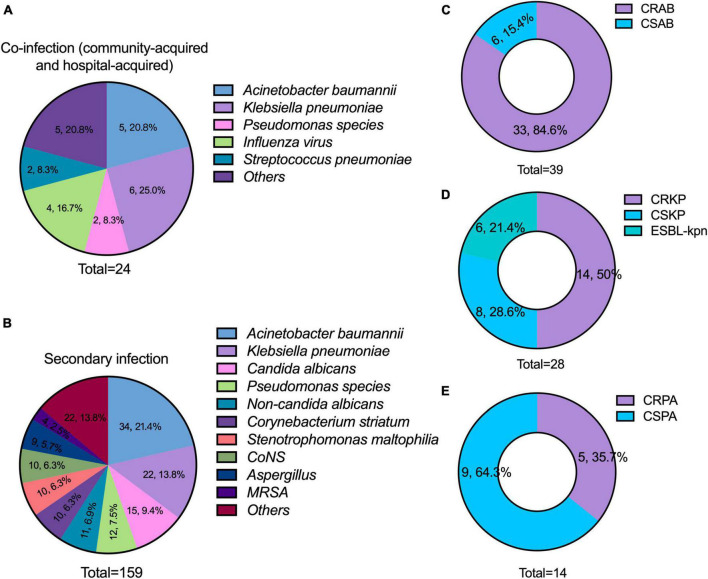
Distribution of pathogens and the results of antimicrobial susceptibility testing. **(A)** Major pathogens of co-infection (including community-acquired and hospital-acquired); **(B)** major pathogens of secondary infection; **(C–E)** incidence of carbapenem-resistant *A. baumannii* (CRAB), carbapenem-resistant *K. pneumoniae* (CRKP), and carbapenem-resistant *P. aeruginosa* (CRPA). CONS, coagulase-negative *Staphylococcus*; MRSA, methicillin-resistant *Staphylococcus aureus*; CSAB, carbapenem-susceptible A. *baumannii*; CSKP, carbapenem-susceptible *K. pneumoniae*; CSPA, carbapenem-susceptible *P. aeruginosa*.

A total of 183 pathogens were isolated from microbiological tests in the 100 patients. The common pathogens for community acquired co-infection were Influenza A virus (50.0%, 4/8), the vast majority were the H3N2 subtype. The common pathogens for hospital acquired co-infection were *A. baumannii* (31.3%, 5/16), *K. pneumoniae* (25.0%, 4/16), and *P. aeruginosa* (12.5%, 2/16) (Figure 2A). The common pathogens for secondary infection were *A. baumannii* (21.4%, 34/159), *K. pneumoniae* (13.8%, 22/159), *Candida albicans* (9.4%, 15/159), and *P. aeruginosa* (7.5%, 12/159) (Figure 2B). There was a high incidence of CR-GNB infection. A total of 84.6% of *A. baumannii*, 50.0% of *K. pneumoniae*, and 35.7% of *P. aeruginosa* were resistant to carbapenems (Figures 2C–E). Particularly, 21.4% of *K. pneumoniae* were extended-spectrum β-Lactamase producers.

### 3.3. The clinical characteristics between the survivors and non-survivors

There were 106 (32.9%) patients who died in the hospital and 216 (67.1%) patients who discharged.

The in-hospital mortality among patients who acquired with secondary pulmonary infection and co-infection was 57.0% (57/100). The overall in-hospital mortality of ICU patients was 48.1% (37/77), and the in-hospital mortality of ICU patients acquired with secondary pulmonary infection and co-infection was 61.4% (27/44).

The distribution of age between the survivors and non-survivors is shown in Figure 1B. The age increased in parallel with death rates (*p* = 0.008). There was no significant difference in the frequency of underlying diseases between the two groups (Table 3). Compared with the survivors, non-survivors had a higher proportion of pulmonary infection (53.8 vs. 19.9%, *p* < 0.001), and more patients received empirical antibiotic therapy (95.3 vs. 77.8%, *p* < 0.001).

**TABLE 3:** Baseline characteristics, complications and prognosis of survivors and non-survivors.

Variables	Total (*n* = 322)	Non-survivors (*n* = 106)	Survivors (*n* = 216)	*p*
Gender, male, *n* (%)	211 (65.5%)	73 (68.9%)	138 (63.9%)	0.377
Age, years, median (IQR)	81.0 (72.0–87.0)	83.5 (75.0–89.0)	79.0 (71.0–86.0)	0.008
ICU admission within 48 h of hospitalization, n (%)	77 (23.9%)	37 (34.9%)	40 (18.5%)	0.001
**Underlying diseases, *n* (%)**				
Hypertension	216 (67.1%)	69 (65.1%)	147 (68.1%)	0.595
Diabetes mellitus	119 (37.0%)	41 (38.7%)	78 (36.1%)	0.654
Chronic cardiovascular diseases	169 (52.5%)	62 (58.5%)	107 (49.5%)	0.131
Chronic respiratory diseases	124 (38.5%)	39 (36.8%)	85 (39.4%)	0.657
Chronic liver diseases	26 (8.1%)	12 (11.3%)	14 (6.5%)	0.134
Chronic kidney diseases	79 (24.5%)	27 (25.5%)	52 (24.1%)	0.784
Cerebrovascular diseases	120 (37.3%)	46 (43.4%)	74 (34.3%)	0.111
Peripheral vascular diseases	82 (25.5%)	28 (26.4%)	54 (25.0%)	0.784
Hemopathy	20 (6.2%)	8 (7.5%)	12 (5.6%)	0.487
Connective tissue diseases	13 (4.0%)	3 (2.8%)	10 (4.6%)	0.639
Peptic ulcer	26 (8.1%)	11 (10.4%)	15 (6.9%)	0.288
Hemiplegia	9 (2.8%)	5 (4.7%)	4 (1.9%)	0.269
Immunocompromised	43 (13.4%)	16 (15.1%)	27 (12.5%)	0.520
Malignancy	87 (27.0%)	29 (27.4%)	58 (26.9%)	0.923
Charlson comorbidity index ≥ 3	191 (59.3%)	69 (65.1%)	122 (56.5%)	0.139
**Exposure history, *n* (%)**				
Smoking	97 (30.1%)	33 (31.1%)	64 (29.6%)	0.782
Drinking	49 (15.2%)	12 (11.3%)	37 (17.1%)	0.173
Vaccination	32 (9.9%)	7 (6.6%)	25 (11.6%)	0.135
Previous glucocorticoid use	24 (7.5%)	7 (6.6%)	17 (7.9%)	0.684
Previous invasive procedure within 3 months before hospital admission	52 (16.1%)	17 (16.0%)	35 (16.2%)	0.970
Empirical use of antibiotics before the first positive culture	269 (83.5%)	101 (95.3%)	168 (77.8%)	<0.001
**Clinical symptoms, *n* (%)**				
Fever	261 (81.1%)	90 (84.9%)	171 (79.2%)	0.217
Sore throat	48 (14.9%)	15 (14.2%)	33 (15.3%)	0.790
Cough	234 (72.7%)	78 (73.6%)	156 (72.2%)	0.797
Sputum	219 (68.0%)	77 (72.6%)	142 (65.7%)	0.212
Fatigue	93 (28.9%)	29 (27.4%)	64 (29.6%)	0.673
Myalgias	33 (10.2%)	12 (11.3%)	21 (9.7%)	0.657
Diarrhea	37 (11.5%)	9 (8.5%)	28 (13.0%)	0.237
Thoracalgia	11 (3.4%)	4 (3.8%)	7 (3.2%)	1.000
Respiratory failure	77 (23.9%)	51 (48.1%)	26 (12.0%)	<0.001
**Laboratory findings, median (IQR)**				
Write blood count × 10^9 per L	6.7 (4.7–9.8)	8.0 (5.2–11.9)	6.2 (4.4–8.9)	0.001
Neutrophil × 10^9 per L	5.2 (3.3–8.5)	6.7 (4.5–10.3)	4.6 (2.8–7.4)	<0.001
Lymphocyte × 10^9 per L	0.6 (0.4–0.9)	0.4 (0.3–0.7)	0.7 (0.5–1.0)	<0.001
Plate count × 10^9 per L	155.0 (111.0–214.0)	134.0 (96.5–193.3)	160.0 (122.0–227.0)	<0.001
Neutrophilic granulocyte percentage	81.0 (69.6–88.7)	88.3 (81.4–93.1)	77.9 (65.8–84.6)	<0.001
lymphocyte percentage	10.2 (5.1–17.3)	5.6 (2.8–11.5)	12.1 (7.0–18.7)	<0.001
Prothrombin time, s	11.9 (11.3–13.0)	12.7 (11.7–14.8)	11.7 (11.2–12.7)	<0.001
Activated partial thromboplastin time, s	31.1 (28.3–34.4)	32.2 (28.1–37.9)	30.9 (28.3–33.3)	0.050
D-dimer, mg/L	0.6 (0.3–1.2)	1.0 (0.4–3.6)	0.5 (0.3–0.9)	<0.001
High-sensitivity cardiac troponin I, ng/L	20.4 (9.6–59.4)	58.4 (21.2–335.8)	13.7 (7.0–29.7)	<0.001
Creatine kinase-MB, mg/mL	1.9 (1.1–3.8)	3.1 (1.6–8.6)	1.6 (0.9–2.5)	<0.001
Creatinine, μmol/L,	91.3 (72.0–158.6)	113.9 (77.0–191.1)	87.6 (66.5–133.8)	<0.001
Lactic dehydrogenase, U/L	271.0 (204.5–384.5)	372.0 (280.5–552.0)	236.5 (187.3–308.8)	< 0.001
Serum ferritin, ng/mL	617.1 (261.6–1053.8)	1048.2 (420.9–1566.0)	583.9 (174.4–770.5)	0.008
IL-6, pg/mL,	49.7 (19.6–124.7)	89.1 (38.7–212.3)	38.5 (14.6–81.7)	<0.001
PCT, ng/mL	0.2 (0.1–0.8)	0.6 (0.2–3.1)	0.1 (0.1–0.4)	<0.001
Hyper C-reactive protein, mg/dL	65.9 (24.5–113.1)	92.5 (55.5–139.8)	46.3 (18.0–98.7)	< 0.001
**Infection**				
Co-infection or secondary infection, n (%)	100 (31.1%)	57 (53.8%)	43 (19.9%)	<0.001
**Critical COVID-19, *n* (%)**	117 (36.3%)	94 (88.7%)	23 (10.6%)	<0.001
**Anti-COVID-19 treatment, *n* (%)**	224 (69.6%)	72 (67.9%)	152 (70.4%)	0.654
Paxlovid	132 (41.0%)	52 (49.1%)	80 (37.0%)	0.039
Azvudine	131 (40.7%)	37 (34.9%)	94 (43.5%)	0.139
**Immunomodulatory treatment, *n* (%)**				
IL-6 or JAK inhibitors	38 (11.8%)	26 (24.5%)	12 (5.6%)	<0.001
Immunoglobulin therapy	33 (10.2%)	23 (21.7%)	10 (4.6%)	<0.001
Glucocorticoid treatment	150 (46.6%)	63 (59.4%)	87 (40.3%)	0.001
Time from onset of symptom to start of glucocorticoid treatment, days, median (IQR)	8.0 (5.0–12.0)	8.0 (5.0–12.0)	9.0 (4.0–12.0)	0.861
Glucocorticoid treatment duration, median, (IQR)	6.0 (3.0–10.0)	5.0 (3.0–9.0)	7.0 (5.0–11.0)	0.013
**Anticoagulant therapy**	167 (51.9%)	65 (61.3%)	102 (47.2%)	0.017
ECMO, *n* (%)	3 (0.9%)	3 (2.8%)	0	0.035
CRRT, *n* (%)	16 (5.0%)	12 (11.3%)	4 (1.9%)	0.001
ICU admission in hospitalization, *n* (%)	97 (30.1%)	47 (44.3%)	50 (23.1%)	<0.001
Length of ICU stay, days, median (IQR)	9.5 (6.0–17.3)	11.0 (6.0–18.0)	9.0 (6.0–15.0)	0.654
Treated with invasive mechanical ventilation	57 (17.7%)	50 (47.2%)	7 (3.2%)	<0.001
Duration of mechanism ventilation, median (IQR)	122.0 (40.0–331.0)	119.0 (41.0–286.8)	130.0 (28.0–569.0)	0.422
Septic shock, *n* (%)	55 (17.1%)	52 (49.1%)	3 (1.4%)	<0.001
Length of hospital stay from exposure to SARS-COV-2, days, median (IQR)	13.0 (7.0–21.0)	10.0 (5.0–17.0)	14.0 (8.0–23.0)	0.001
Length of hospital stay, median (IQR)	16.0 (8.0–29.0)	14.0 (6.0–28.0)	18.0 (10.0–29.8)	0.012

IQR, range interquartile; IL-6, interleukin-6; PCT, procalcitonin; JAK, Janus kinase; ECMO, extracorporeal membrane oxygenation; CRRT, continuous renal replacement therapy.

Abnormal laboratory parameters were commonly seen in the non-survivors. A total of 88.7% of patients classified as critical COVID-19 were died. Three patients were treated with extracorporeal membrane oxygenation (ECMO), and none survived. Death was associated with patients who received IL-6 or JAK inhibitors, systematic corticosteroids and intravenous immunoglobulin. The patients admitted to the ICU (23.1 vs. 44.3%, *p* < 0.001) during hospitalization, and suffered from septic shock (1.4 vs. 49.1%, *p* < 0.001) had a high mortality.

### 3.4. The causes of death

**FIGURE 3:**
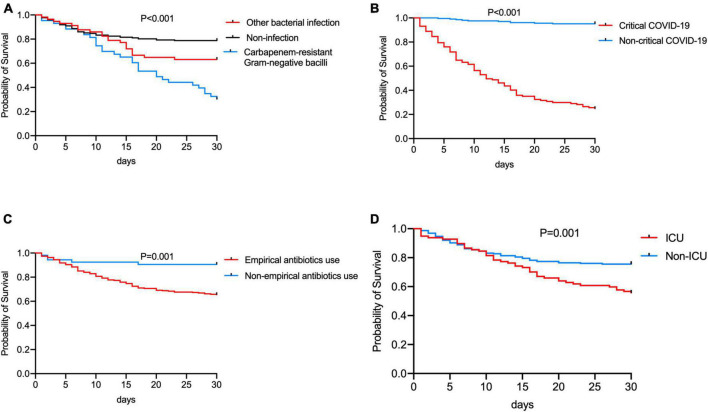
Comparation of the 30-day Kaplan-Meier survival curve between patients **(A)** infected by Carbapenem-resistant Gram-negative bacilli, other bacteria and non-infection, **(B)** with/without critical COVID-19, **(C)** with/without receiving empirical antibiotic treatment, and **(D)** with/without admitting to ICU in hospitalization.

The survival time of the non-survivors was analyzed. The distribution of survival time from the symptoms onset to death showed two peaks, with the first one at approximately 14 days, and the second one at approximately 26 days (Figure 1C). The causes of death were analyzed (Figure 1D), the most common cause of death was septic shock, followed by respiratory failure. The risk factors for significantly higher 30-day mortality (*p* ≤ 0.001) were summarized in Figures 3A–D, patients who were infected with CR-GNB and other bacteria, classified as critical COVID-19, received empirical antibiotic treatment and admitted to ICU during hospitalization had a high 30-day mortality.

## 4. Discussion

Our study confirmed that in a significant proportion of cases, secondary bacterial, fungal or viral pulmonary infection and co-infection could complicate the hospital course in elderly COVID-19 patients. In this study, we found the incidence of co-infection was 7.5%, and the incidence of secondary infection was 27.3%, which was slightly higher than previously published results (Lansbury et al., 2020; Ripa et al., 2021). The incidence of secondary pulmonary infection and co-infection among patients admitted to ICU was 50.6 and 13.0%, respectively, which was higher than previous study [early infection (the same definition as co-infection in this article) was 8.7%, late infection (the same definition as secondary infection in this article) was 41.1%] reported in ICU patients (Pandey et al., 2022). The possible reason was that we focused on older people. Another study reported that 72.7% of the COVID-19 ICU patients had secondary infection (Akrami et al., 2023), which was higher than reported in this study. The possible reason might be due to widespread usage of mechanical ventilator that expose patients to infection.

Previous studies have identified critical COVID-19, oxygen saturation ≤94%, ferritin levels <338 ng/mL, PCT > 0.2 ng/mL, early need for ICU admission, respiratory failure, and severe lymphopenia as independent risk factors for secondary pulmonary infection and co-infection in COVID-19 patients (Feng et al., 2020; Ripa et al., 2021; Moreno-García et al., 2022). Our study showed some similar results. We identified predictors, including ICU admission within 48 h of hospitalization, cerebrovascular diseases, critical COVID-19, and PCT > 0.5 ng/mL. The proportion of critical cases in elderly patients with COVID-19 was high, most patients needed to be transferred to ICU for treatment. The high rate of invasive mechanical ventilation and central catheter placement in ICU may increase secondary bacterial, fungal or viral pulmonary infection and co-infection, resulting in high mortality. Previous study showed immune responses, such as lymphocyte count, T cells, were substantially decreased in COVID-19 patients, which made them at a high risk of infection (Lin et al., 2020). In the case of COVID-19, early detection of patients who are at high-risk of secondary bacterial or fungal pulmonary infection and co-infection can help guide management decisions and effectively combat this deadly respiratory pandemic.

Inflammatory biomarkers may help in early diagnose of secondary bacterial or fungal pulmonary infection and co-infection in COVID-19 patients (Wang L. et al., 2020). Despite the overlap of viral and bacterial symptoms, some typical biomarkers that indicate bacterial infection can still be found, including decreased lymphocyte count, elevated white blood cell count, neutrophil count, IL-6, PCT, and hyper C-reactive protein. Elevated PCT is often used as a biomarker for bacterial infections, within the normal range in viral infections (Schuetz et al., 2011). In this study, we found PCT > 0.5 ng/mL could predict secondary bacterial pulmonary infection and co-infection. This was consistent with previous result (Malik et al., 2021). Lymphocytes play a key role in the adaptive immunity against viral infection (Wang F. et al., 2020). COVID-19 patients often developed lymphocytopenia, the immunodeficient state that made patients more vulnerable to be infected with other respiratory pathogens. In this study, we found patients with secondary pulmonary infection and co-infection had lower lymphocyte counts than patients without infection. Several studies also showed that one of the typical characteristics of COVID-19 patients was lymphocytopenia, which was significantly associated with poor prognosis (Huang and Pranata, 2020; Yang et al., 2020).

Previous study identified the common bacteria in co-infection were *M. pneumonia*, *P. aeruginosa*, and *H. influenzae* (Lansbury et al., 2020), it was different from this study, the most common pathogen of community acquired co-infection was Influenza A virus. The high incidence of Influenza A virus may be a dual seasonal pattern in China, with the prevalence in northern China following a winter pattern (Shu et al., 2010). The common pathogens of hospital acquired co-infection and secondary infection were *A. baumannii*, *K. pneumoniae*, and *P. aeruginosa*, which were consistent with common pathogens frequently identified in hospital acquired pneumonia (Jones, 2010). The results of antimicrobial susceptibility testing showed that most of *A. baumannii* and *K. pneumoniae* were resistant to carbapenems, the patients infected with CR-GNB had a considerably high mortality. Previous study showed in a COVID-19 respiratory sub-intensive care unit, carbapenem-resistant *A. baumannii* (CRAB) infections occurred in almost half of patients and were associated with high mortality (Iacovelli et al., 2023). Thus, these findings suggested that infection control measures should be enhanced to prevent the transmission of CR-GNB in healthcare settings among older patients. Firstly, routine infection control measures including environmental cleaning, staff education, hand hygiene, microbiological capacity need to be strengthened; Secondly, identification of “at-risk” carriers of CR-GNB should be performed on admission, pre-emptively isolated in a single room; Thirdly, active screening for CR-GNB by obtaining swabs from rectal or perirectal areas, and any other site that is either actively infected or colonized. If the screening test result is positive for CR-GNB, patient isolation and contact precautions are continued. Finally, antimicrobial stewardship should be implemented. Antibiotic usage is a primary driver of antibiotic resistance. Previous study showed that antibiotic exposure (cephalosporins, fluoroquinolones, and carbapenems) was a risk factor for CR-GNB infection (Çölkesen et al., 2023).

In our study, the majority of patients (83.5%) received empirical antibiotic therapy before the first positive culture result, which was consistent with a previous study (60.0–100%) (Chong et al., 2021). The reason for the overuse of antibiotics was the difficulty in early identification of bacterial or fungal co-infections. Moreover, the possibility of secondary bacterial or fungal pulmonary infection should be considered. In this study, the main empirical antibiotic prescription was carbapenems, followed by cefoperazone/sulbactam, cephalosporin, moxifloxacin, and piperacillin tazobactam. In particular, 124 patients received empirical therapy with more than one antibiotic. However, overuse of antibiotics can lead to emergence of MDR pathogens. Implementing strategies, such as empirical therapy should take into account local epidemiological data and/or individual risk factors, in-time de-escalation of antibiotic therapy according to culture data, using high-dose, susceptible antibiotics, are essential in appropriate antibiotic usage. A variety of studies have demonstrated that optimizing antimicrobial use could improve patient safety, minimize antimicrobial resistance, reduce the side effects of antibiotics, decrease the usage of unnecessary antibiotics, shorten hospital stay, and cut down economic cost (Owens, 2009; Campion and Scully, 2018; Majumder et al., 2020). The COVID-19 guidelines recommended that antibiotics should be considered for patients who may only have bacterial infection, comorbidities, or at high risk of complications from untreated bacterial infection (Poston et al., 2020).

In the meta-analysis of clinical trials of patients hospitalized for COVID-19, administration of IL-6 antagonists, compared with placebo or usual care, was associated with lower 28-day all-cause mortality (Shankar-Hari et al., 2021). In this study, a small number of patients were treated with IL-6 antagonists, and we did not find that the use of IL-6 antagonists could significantly improve patients’ survival. There was inconsistency between different studies. More patients received IL-6 antagonist treatment in this study acquired bacterial or fungal pulmonary infection, the infected patients had high mortality. In addition, the association of IL-6 antagonists with lower 28-day all-cause mortality was significant among patients who did not require invasive mechanical ventilation at randomization (Shankar-Hari et al., 2021). However, most patients received invasive mechanical ventilation treatment in this study. The patients received systematic corticosteroids and intravenous immunoglobulin had a high mortality. There were some possible reasons. Firstly, more severe patients were treated with these treatments; Secondly, patients received systematic corticosteroids and intravenous immunoglobulin may predispose to secondary bacterial or fungal pulmonary infection and co-infection. Emerging evidence has unveiled infection as one of the mortal causes of post-SARS-CoV-2 infection (Patton et al., 2023). Thirdly, these treatments may be associated with declined immune responses. Owing to relatively small sample size and retrospective nature of this study, there may also be a selection bias when identifying factors that influence the clinical outcomes. A larger cohort study of patients with COVID-19 pneumonia are needed to further explore.

In this study, we did not find that treatment with paxlovid could improve patients’ survival. Previous study reported that paxlovid was highly effective in reducing the risk of severe COVID-19 or mortality in the era of Omicron and in real world setting (Najjar-Debbiny et al., 2023). The reasons for the inconsistent results may be the majority of patients treated with paxlovid in this study were severely or critically ill, and more likely to develop secondary or co-occurring bacterial, fungal, and viral infections. Considering older patients have more basic diseases, and there is a special situation of multi-drug sharing, paxlovid has a high potential to cause clinically important drug–drug interactions with other concurrent medications. In many settings, paxlovid will be primarily prescribed by practitioners who may lack in-depth knowledge of managing these complex drug–drug interactions associated with paxlovid, leading to unintended denial of therapy or the occurrence of serious adverse events. However, these results should be interpreted with caution owing to potential bias and residual confounding in this observational study. Double-blinded randomized clinical trials should be conducted to validate these results.

In this study, the overall in-hospital mortality of COVID-19 patients was 32.9%, and the in-hospital mortality of patients who acquired secondary pulmonary infection or co-infection was 57.0%. The mortality was higher than previous study (Zhou et al., 2020), the possible reason was that our study only focused on elderly patients. Additionally, we found that patients with bacterial, fungal or viral pulmonary infections had increased rates of ICU admission and prolonged hospital stay. The overall in-hospital mortality of ICU patients was 48.1%, and the in-hospital mortality of ICU patients acquired with secondary pulmonary infection and co-infection was 61.4%. Previous study showed the mortality among adults with COVID-19 admitted to the ICU who acquired secondary infection was 83% (Pourajam et al., 2022). The mortality was higher than in this study. The possible reason was that more ICU patients were infected with CR-GNB in the previous study. In this study, the mortality of patients infected by CR-GNB was 76.9%. The result was much closer. However, this was unadjusted to baseline patient characteristics and cannot be completely attributed to bacterial, fungal or viral pulmonary infections.

There are some limitations in this study. First, this is a single-center retrospective study, the results of our analysis are limited to short-term follow-up, and the long-term effects of infection on these patients are unknown. Second, not all patients with suspected infection had ordered microbiological testing due to unprecedented circumstances and immense pressure on hospital systems during the COVID-19 pandemics. However, the results of this study are still representative and reflect the real-world situation of elderly COVID-19 patients in China during the pandemics.

## 5. Conclusion

We described the incidence and predictors of secondary bacterial, fungal or viral pulmonary infection and co-infection in elderly COVID-19 patients during the Omicron BA.7 and BA.5.2 variant pandemics in a Chinese tertiary general hospital, showing a high burden of infection caused by CR-GNB. The inflammatory biomarker PCT > 0.5 ng/mL played an important role in the early prediction of secondary bacterial pulmonary infection and co-infection in COVID-19 patients, this could provide a reference for the rational use of antibiotics.

## Data availability statement

The raw data supporting the conclusions of this article will be made available by the authors, without undue reservation.

## Ethics statement

The studies involving humans were approved by the Ethics Committee of Peking University First Hospital, Beijing, China (Approval No. 2023-yan-090). The studies were conducted in accordance with the local legislation and institutional requirements. The Ethics Committee/Institutional Review Board waived the requirement of written informed consent for participation from the participants or the participants’ legal guardians/next of kin because Due to the retrospective observational study and the confidentiality of patient information.

## Author contributions

CZ: Formal analysis, Data curation, Writing – original draft. YJ: Writing – review and editing, Data curation. LS: Data curation, Writing – review and editing. HL: Conceptualization, Writing – review and editing. XL: Conceptualization, Funding acquisition, Writing – review and editing. LH: Data curation, Funding acquisition, Writing – review and editing.

## Footnotes

1
https://covid19.who.int/

2
http://www.nhc.gov.cn/
